# Valacyclovir reduces inflammation and IgG subclass activation by suppressing herpesvirus reactivation in antarctic expeditioners

**DOI:** 10.3389/fimmu.2026.1957541

**Published:** 2026-10-19

**Authors:** Douglass M. Diak, Sara Bustos-Lopez, Cody L. Gutierrez, Brian E. Crucian, Satish K. Mehta

**Affiliations:** 1 Immunology and Virology Laboratory, NASA-Johnson Space Center (NASA-JSC), Aegis Aerospace, Houston, TX, United States; 2 Immunology and Virology Laboratory, NASA-JSC, JES Tech, Houston, TX, United States; 3 Immunology and Virology Laboratory, NASA-JSC, KBR, Houston, TX, United States; 4 Immunology and Virology Laboratory, NASA-JSC, NASA, Houston, TX, United States

**Keywords:** Antarctica, CRP, herpes viruses, immunoglobulin, spaceflight, valacyclovir

## Abstract

**Introduction:**

Astronauts experience a higher frequency and greater magnitude of latent herpesvirus reactivation than the general population during and after space travel. This is likely due to space-related physiological and psychological stressors. Repeated symptomatic or asymptomatic viral reactivations may further increase immune burden and contribute to persistent low-grade inflammation and immune dysregulation, highlighting the need for effective countermeasures during long duration missions. In our previous randomized, placebo-controlled trial in Antarctic expeditioners, we demonstrated that daily administration of valacyclovir (1g/day) markedly reduced both the frequency and load of Epstein-Barr virus (>24fold) and herpes simplex virus-1 (>4fold). Using the saliva supernatants from this published parent study, the present brief research report examines salivary C-reactive protein (CRP) and immunoglobulins A, G1–4, and M to further characterize downstream immune effects of antiviral suppression.

**Methods:**

Detailed participant information, saliva sampling procedures, and the study timeline are described in the previous manuscript. Residual frozen saliva supernatants from the original study were analyzed for CRP (Salimetrics) and immunoglobulins IgA, IgM, and IgG1–4 (Millipore Sigma), according to manufacturer instructions.

**Results:**

CRP concentrations were significantly lower in the valacyclovir group compared with the placebo group across the mission. IgG subclasses demonstrated clear treatment effects: IgG1, IgG3, and IgG4 were reduced in the valacyclovir group, while IgG2 remained unchanged. IgA and IgM showed no overall differences between groups.

**Discussion:**

Suppressing latent herpesvirus reactivation with valacyclovir reduces inflammatory and humoral immune activation in Antarctic expeditioners. These findings advance the understanding of virus-driven immune dysregulation and support antiviral prophylaxis as a potential countermeasure for maintaining immune stability during long-duration spaceflight.

## 1 Introduction

Prolonged spaceflight exposes astronauts to physiological and psychological stressors that can promote the reactivation of latent herpesviruses, including varicella-zoster virus (VZV), herpes simplex virus-1 (HSV-1), and Epstein-Barr virus (EBV). It is hypothesized that viral reactivation is a result of immune dysregulation due to a) chronic elevation of stress hormones [e.g., cortisol) triggered and sustained by spaceflight stressors and b) the direct negative impact of microgravity on immune cell structure and function (see Winer et al., 2025 for an in-depth review of “astroimmunology” (1)].

Viral shedding is readily detected by qPCR in the saliva of crewmembers onboard the International Space Station (ISS) (2), with a select few who have occasionally presented clinically during missions onboard ISS (3, 4). Repeated reactivations, whether asymptomatic or symptomatic, are not immunologically benign. Rather, each reactivation episode pulses the immune system with rapid CD8+ T cell and NK cell cytotoxicity engagement, antibody production and neutralization from plasma cells, and coordinated-cytokine release from a myriad of cells to signal danger and contain the spread of the virus. While essential, these processes result in the expansion of the viral-specific effector memory T cells, memory-like NK cells, and pre-existing class-switched memory B cells, leading to a state of memory inflation and a reduction in memory pool diversity, consuming immune resources and elevating overall baseline inflammation (5–7). This additional viral burden compounds the immune stress already present from spaceflight and may increase long-term risks for infection, cancer, and autoimmunity in astronauts. These combined effects highlight the need for directed countermeasures that prevent latent viral reactivation and help maintain crew health.

Prior to implementation of any countermeasure in spaceflight, high-fidelity ground analogs are utilized first to evaluate feasibility, efficacy, and potential attrition of countermeasure strategies. Living and working at Antarctica stations provides astronaut-like–though generally milder– physiological and psychological stressors that influence immune function. Factors such as isolation and confinement, circadian misalignment, extreme environmental conditions, and the unique constraints of station life overlap closely with conditions experienced during space missions. Terrestrial studies in Antarctica have shown similar patterns of stress-induced immune alterations, including suppression of innate immune functions (8), changes in inflammatory responses (9) and subsequent reactivation of latent herpesviruses such as EBV, HSV-1/2 and HHV-6 (10, 11).

In line with these established analog characteristics and in an effort to identify potential antiviral countermeasures for spaceflight, we evaluated subjects receiving prophylactic valacyclovir (1g/day) during a 6–8 month winter-over trial at the McMurdo and South Pole Stations in Antarctica. Crew studies in this habitat have been documented as valid spaceflight ground analogs for immune dysregulation and viral reactivation (9, 12, 13). Findings from our previous study demonstrated significant reductions in EBV (~24-fold) and HSV-1 (~5-fold) shedding with daily valacyclovir treatment, compared with placebo (11). Preliminary results on saliva samples showed reduced levels of inflammatory cytokines, suggesting that reduced viral expression through valacyclovir may help stabilize immune function and limit inflammation.

As a corollary to this study, residual frozen saliva supernatants from the study were analyzed to quantify salivary C-reactive protein (CRP) and immunoglobulins (IgA, IgM, and IgG1–4). The goal was to provide a more detailed view of inflammatory and mucosal humoral responses associated with antiviral suppression of latent herpesvirus reactivation under extreme environmental stress. The following is a brief report documenting the additional findings.

## 2 Methods

Please refer to our previous manuscript for participant information, study design, and initial analyses at the following link: https://www.frontiersin.org/journals/virology/articles/10.3389/fviro.2023.1157659/full

### 2.1 CRP and Ig assays

To limit additional freeze-thaw cycles, CRP and Ig analyses were run on the same day for each sample. Saliva supernatants were removed from the −80 °C freezer and allowed to thaw at room temperature. Samples were then aliquoted for either the CRP (150µl) or Ig (100µl) protocols and assayed immediately. Tests for CRP (pg/mL) were performed per the manufacturer’s instructions using the Salimetrics Salivary C-Reactive Protein Enzyme Immunoassay Kit (catalog#: 1-2102, Salimetrics, LLC, Carlsbad, CA, USA). Absorbance at 450 nm was read using a Tecan Spark plate reader (Tecan, Morrisville, NC, USA).

For the immunoglobulin assay, a protease inhibitor cocktail (catalog#: p2714, Sigma Aldrich, St. Louis, MO, USA) was added at 1:500 of the saliva supernatant sample, which was then centrifuged at 10, 000 x g for 10 minutes to remove any freeze-thaw particulates or aggregates. The supernatant was collected and diluted 1:2 with assay buffer—this method improves analyte recovery and reduces the chance of bead aggregation when using saliva samples for bead-based milliplex kits, nominally used for serum or tissue culture supernatants. The multiplex test for IgA, IgM, and IgG1-4 (ng/mL) was then performed using the diluted samples per the manufacturer’s instructions using the MILLIPLEX^®^ Human Immunoglobulin Isotyping Panel (catalog#: HGAMMAG-301K, Millipore Sigma, Burlington, MA, USA) on a Luminex MAGPIX instrument (DiaSorin, Austin, TX, USA).

All samples were assayed in duplicate, and the average of the duplicates was used in the statistical analyses.

### 2.2 Data analysis

Unpaired t-tests were used to detect overall significant differences in the total concentration of CRP, IgA, IgM, IgG1, IgG2, IgG3, and IgG4 between the valacyclovir and placebo participants at both stations across the entire mission in Antarctica. A mixed-effects model was then applied to evaluate main effects of time, treatment group, and their interaction. This model was used to assess within-group changes with comparisons to all months, as well as between-group differences at each monthly timepoint. Tukey’s test was use for multiple-comparison correction. Statistical significance was defined as p < 0.05. Analyses were conducted in GraphPad Prism v10.2.0 (GraphPad Software, LLC, Boston, MA, USA).

## 3 Results

### 3.1 Residual sample numbers

The original study included a total number of 1, 054 saliva samples collected from 32 subjects. This continuation study analyzed a total of 1, 044 saliva samples (624/625 placebo; 420/429 valacyclovir). The samples not included were due to insufficient volume remaining from prior analyses.

### 3.2 CRP (pg/mL)

The mean level of CRP was significantly lower in the valacyclovir group (660 ± 36) compared to the placebo group (1, 472 ± 39) across the mission (p < 0.0001) (**Figure 1A**). Significant group differences were observed at all monthly timepoints except October (South Pole only) (**Figure 1B**). This difference was consistent at both the South Pole and McMurdo stations (**Figures 1C, D**). Station-specific month-by-month CRP plots are shown in **Supplementary Figure S1**, revealing a consistent decrease in CRP across all mission time points at both stations.

**Figure 1:**
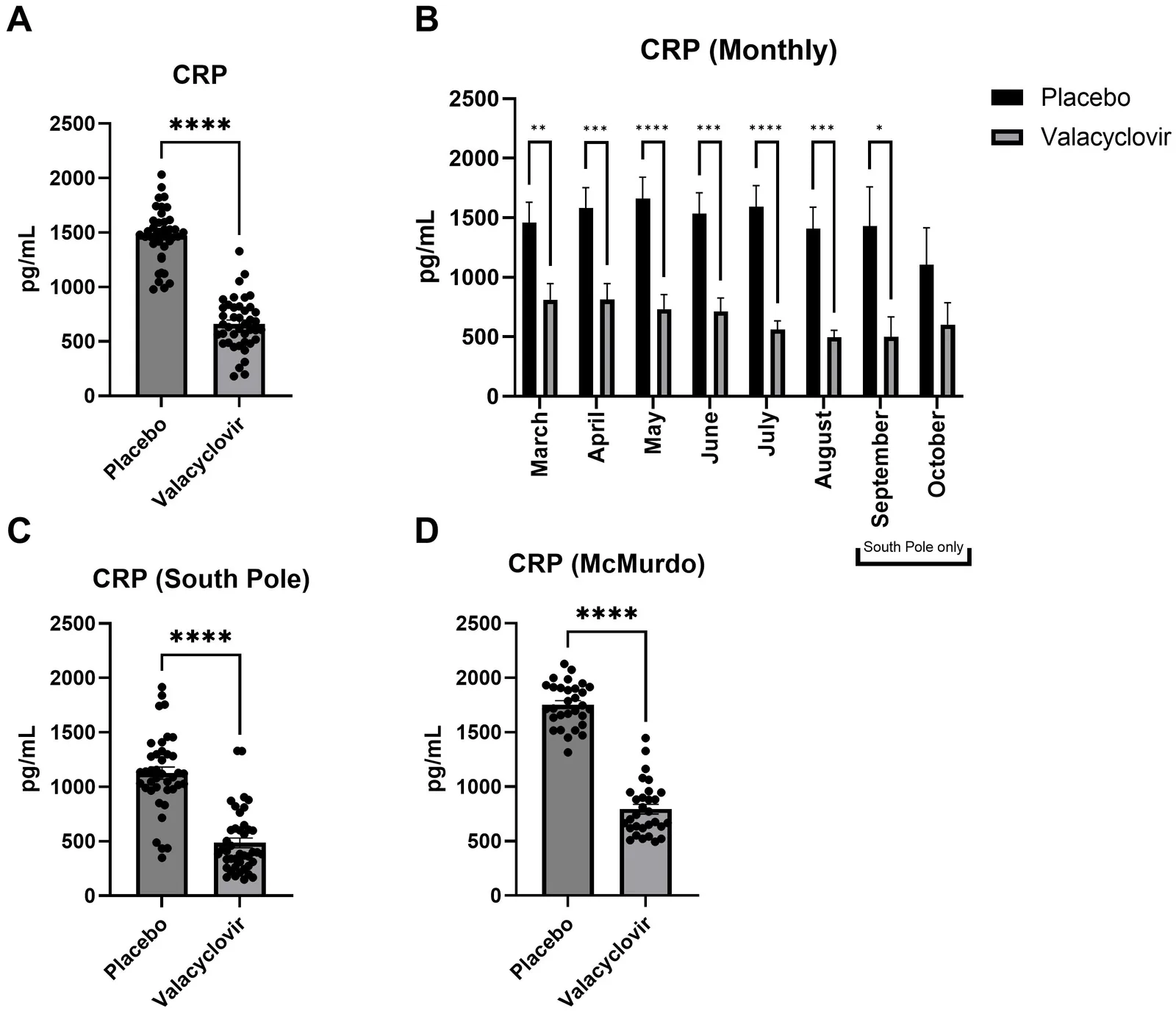
Salivary CRP measurements in placebo and valacyclovir groups. Overall, monthly, and station-specific CRP concentrations are shown for combined and separate station cohorts. *p < 0.05, **p < 0.01, ***p < 0.001, ****p < 0.0001.

### 3.3 Immunoglobulins (ng/mL)

#### 3.3.1 IgA

Overall, for IgA, no significant difference was observed between groups across the mission (placebo: 3, 666 ± 120 vs. valacyclovir: 3, 689 ± 126; p = 0.8948) (**Figure 2A**). Combined station data did reveal a significant increase in IgA for the valacyclovir group in March (placebo: 2, 968 ± 217 vs. valacyclovir: 4, 167 ± 337; p = 0.0011) (**Figure 2B**). Station-specific analysis showed opposing trends: IgA was significantly lower at the South Pole station (p < 0.05) for the valacyclovir group (**Supplementary Figure S2A**), while being significantly higher at McMurdo (p < 0.0001) (**Supplementary Figure S2C**). In September, IgA was significantly reduced for the valacyclovir group at the South Pole station (S2B), whereas significant increases of IgA were found at McMurdo in March and July (**Supplementary Figure S2D**). Station level and monthly IgAs are shown in **Supplementary Figures S2A–D**.

**Figure 2:**
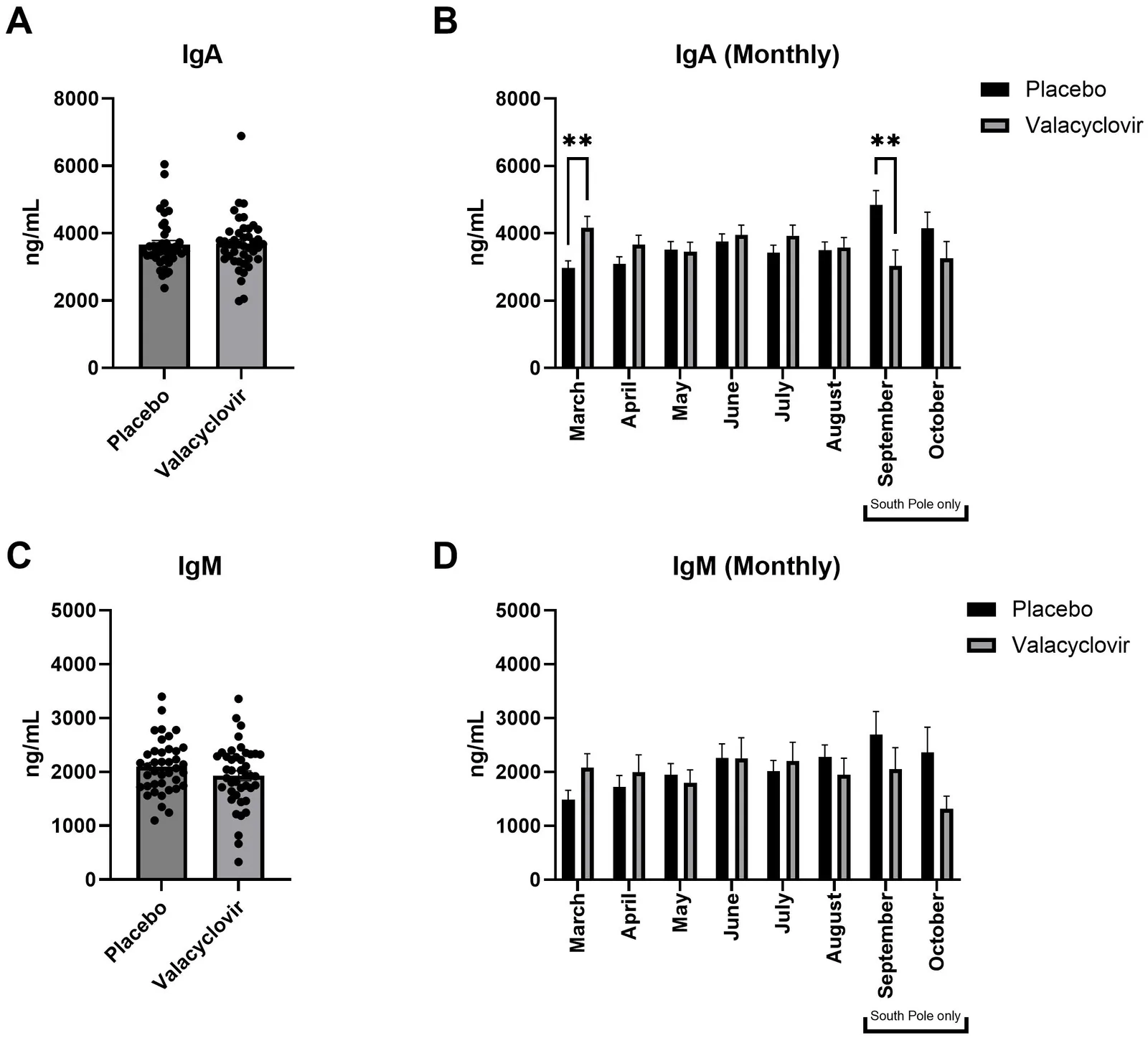
Salivary IgA and IgM measurements. Overall and monthly IgA and IgM concentrations are shown for placebo and valacyclovir groups across combined stations. **p < 0.01.

#### 3.3.2 IgM

No difference was observed for IgM between groups when combining station data nor when combining longitudinally by month (**Figures 2C, D**). Assessment at the individual stations (**Supplementary Figures S2E–H**) shows that IgM was significantly reduced in the valacyclovir group at the South Pole (placebo: 2, 019 ± 100 vs. valacyclovir: 1, 603 ± 101; p < 0.01) (**Supplementary Figure S2E**). Longitudinal analysis within placebo groups revealed a significant increase in IgM in September compared to March and April (**Supplementary Figure S2F**). No significant differences were observed at McMurdo (**Supplementary Figures S2G, H**). Station-level and monthly IgMs are shown in **Supplementary Figures S2E–H**.

#### 3.3.3 IgG1, IgG2, IgG3, IgG4

IgG1 was significantly lower in the valacyclovir group (6, 469 ± 410) than in the placebo group (8, 780 ± 411) across the mission (p < 0.0001) (**Figure 3A**), driven exclusively by the findings at McMurdo (**Supplementary Figure S3D**), and IgG2 showed no overall difference (placebo: 26, 311 ± 1, 396 vs. valacyclovir: 23, 348 ± 1, 186; p = 0.1085) (**Figure 3B**). Station-specific analysis showed higher IgG2 in the valacyclovir group at South Pole (p < 0.05) (**Supplementary Figure S3G**) but lower IgG2 in the same group at McMurdo (p < 0.001) (**Supplementary Figure S3I**). No monthly differences were observed. IgG3 was significantly lower in the valacyclovir group (171.9 ± 6.42) than in the placebo group (190.7 ± 5.83) overall (p < 0.05) (**Figure 3C**), with no station-specific or monthly differences (**Supplementary Figures S3K–O**). IgG4 was significantly lower in the valacyclovir group (380.2 ± 10.25) than in the placebo group (446.4 ± 9.14) overall (p < 0.0001) (**Figure 3D**), driven by the findings at the McMurdo (mean difference: −61.35 ± 12.41; p < 0.0001) and the South Pole stations (mean difference: −51.69 ± 20.36; p < 0.05) (**Supplementary Figures S3Q, S**). Notably, 65% of IgG4 data points were outside the assay detection range (673 above, 2 below, 371 within). Excluding these out-of-range values did not materially change group results (data not shown). **Supplementary Figures S3A–T** provide corresponding month-stratified and station-specific IgG1–IgG4 profiles.

**Figure 3:**
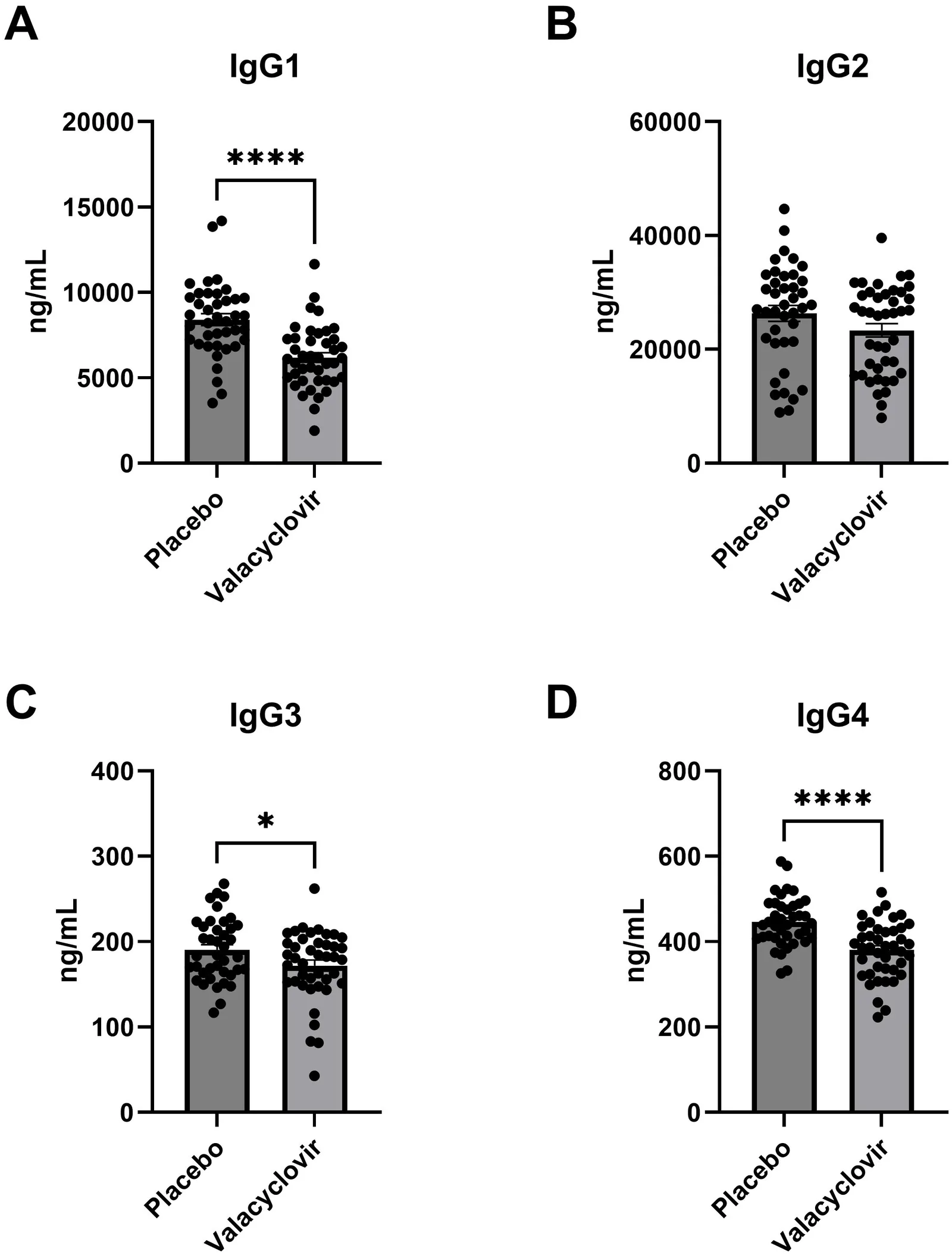
Salivary IgG subclass measurements. Overall IgG1, IgG2, IgG3, and IgG4 concentrations are shown for placebo and valacyclovir groups. *p < 0.05, ****p < 0.0001.

### 3.4 Consolidated visualization

**Figure 4** provides a consolidated forest plot of mean differences (valacyclovir – placebo) with 95% confidence intervals (CIs) for all analytes, offering a cross-analyte and cross-station comparison that complements the individual results shown in **Figures 1**–**3**.

**Figure 4:**
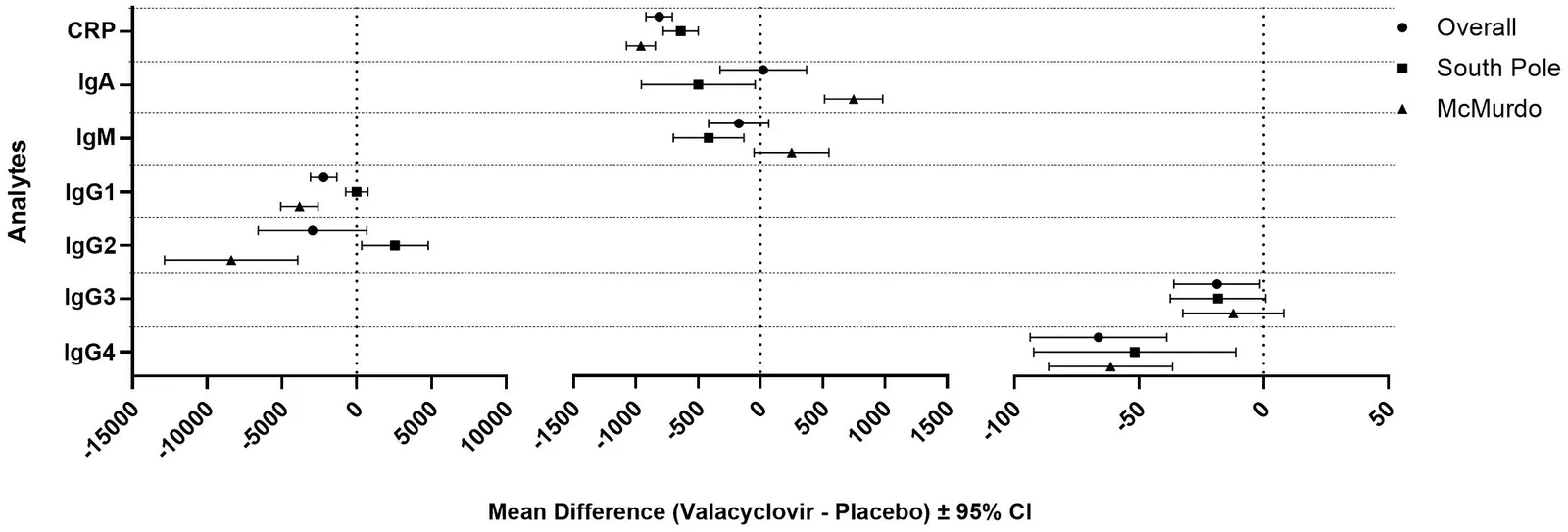
Forest plots for overall and station-specific treatment effects across all analytes. Mean differences (valacyclovir – placebo) with 95% confidence intervals are shown and separated by large, medium, and small-scale analytes.

## 4 Discussion

Residual saliva supernatants from our previous study afforded the opportunity to conduct additional analyses on the effects of daily valacyclovir treatment in the Antarctic expeditioners. The initial study proposed that the lower cytokine levels in the valacyclovir group reflected reduced “viral burden”, as expeditioners receiving daily antivirals experienced less EBV and HSV-1 reactivation in both viral load and frequency than those taking placebo. Although these results were promising, the immunological consequences of the substantial herpesvirus reactivation observed in the placebo group were not fully characterized. In this follow-up study, we analyzed all remaining saliva samples to assess systemic innate inflammation via CRP and adaptive humoral responses through IgA, M, and G1–G4.

Valacyclovir treatment produced a significant reduction in systemic inflammation, demonstrated by a greater than twofold decrease in salivary CRP compared with placebo. Nearly every month of deployment showed clear group differences, and CRP in the valacyclovir group exhibited a downward trend over time. These findings are consistent with our previously published findings showing reduced pro-inflammatory cytokines in the same cohort (11), supporting the hypothesis that chronic low-grade inflammation in the placebo group was largely driven by recurrent herpesvirus reactivation. Elevations in CRP are well documented in the context of repeated viral events, including long COVID and Human Herpes Virus-6 (14), as well as with EBV and CMV (15). Normal ranges for salivary CRP have been previously determined and provide a useful reference point for comparison of our results in this spaceflight ground analog. Desai and Mathews (16) reported salivary CRP concentrations of 35–217 pg/mL in healthy adults (aged 20–35) using passive drool collections. Another study found that the mean CRP levels in saliva of healthy human volunteers (aged 20–70) was 285 pg/ml compared to cardiac patients (aged 43–86) at 1, 680 pg/ml (17). In contrast, expeditioners (aged 27–63) in this study exhibited CRP levels far above the normative values, closer to those with clinical cardiac issues, with levels in the placebo group ranging from 977.4–2, 032 pg/mL and CRP levels in the valacyclovir group ranging from 180.6–1, 329 pg/mL. These findings indicate that valacyclovir markedly reduced inflammatory burden via the suppression of EBV and HSV-1, bringing some individuals closer to normal physiological ranges.

While blood samples were not taken in the original study, salivary immunoglobulin measurements may offer insight into systemic serum titers because IgG, IgM, and IgA can enter the oral cavity through passive paracellular leakage across the gingival crevice (18). In our study, there were no overall differences in IgA and IgM between the two study groups and only minimal monthly variations. The absence of significant overall differences in IgA and IgM between treatment groups is expected. IgA, the predominant mucosal immunoglobulin, provides continuous protection against external pathogens, while IgM is primarily involved in early responses to novel antigen exposure. In primary infectious mononucleosis, patients normally present with strong levels of EBV-IgM first, followed by a gradual decline and a subsequent increase in IgG antibodies to viral capsid antigens and early lytic proteins (19). Because the conditions of this study (isolation in Antarctica) promoted internal latent herpesvirus reactivation rather than new externally sourced infections, these analytes were not anticipated to vary substantially between groups.

In contrast, the IgG subclass data provides strong evidence of valacyclovir’s indirect impact on adaptive immunity. IgG1, IgG3, IgG4 were all significantly reduced in the valacyclovir group, while IgG2 remained unchanged. This pattern is consistent with the current understanding of IgG subclasses. IgG2 antibody production is normally associated with responses to polysaccharides, making them the primary humoral response to bacterial infections (20, 21); thus, equivalent values of IgG2 between the groups is unsurprising. IgG1 and IgG3 are more dominant against viral infections and tend to work in concert to dampen viral responses with IgG3 appearing first in the course of infection, followed by an abundance of IgG1 (22). Furthermore, recent research into IgG subclasses and EBV has revealed that IgG1 normally dominates the latent viral antibody response while IgG3 dominates when the virus reactivates (23). IgG3 also plays a significant role in neutralizing the ability of HSV-1 to evade the immune response (24). Therefore, the significant reductions in IgG1 and IgG3 in the valacyclovir group are consistent with reduced herpesvirus reactivation.

IgG4 also decreased in the valacyclovir group in our data set, an unexpected result for a spaceflight ground analog of extreme environment and quasi-isolation. IgG4 is most commonly associated with allergic responses (in coordination with IgE and Th2 responses to allergens) and/or fighting off parasitic infections (25), neither of which is likely in this setting. However, in our previous study, we showed that the placebo group had elevated levels of herpes viral shedding and substantially more IL-10, a potent anti-inflammatory cytokine to suppress immune activation. We hypothesized that IL-10 could arise from human or viral sources, as EBV and HSV-1 can induce production of IL-10 to evade immune surveillance by largely dampening T-cell mediated antiviral responses (26). Interestingly, IL-10 is also known to act directly on B cells to promote IgG4 class-switch recombination (CSR) (27). This finding, in conjunction with recent evidence that EBV reactivation in peripheral blood mononuclear cell cultures induces IgG4 production (28), provides a reasonable explanation for the reduced IgG4 we observed: valacyclovir decreases herpesvirus reactivation, which leads to a reduction in IL-10 exposure and CSR, which leads to a decrease IgG4 production.

Interestingly, when comparing results at South Pole and McMurdo, we observed opposing trends in the immunological effects of valacyclovir across IgA, IgM and IgG2 (**Figure 4**). Valacyclovir produced positive mean differences in IgA and IgM at McMurdo and negative differences at South Pole, while IgG2 showed the opposite pattern with higher valacyclovir–placebo differences at the South Pole. These station-specific differences likely reflect how each station’s environment shapes mucosal and systemic immune responsiveness. We also observed station-specific patterns in our parent study, where valacyclovir suppressed EBV and HSV-1 reactivation far more strongly at the South Pole than at McMurdo, consistent with differences in station location (coastal vs inland) and degree of isolation. McMurdo functions more like a small Antarctic town, with larger populations (up to 200 during winterover), more frequent resupply, and greater interpersonal contact (29). In such an environment, even modest reductions in viral activity from valacyclovir may be sufficient to produce detectable increases in IgA and IgM. Conversely, the Amundsen-Scott South Pole station is a highly isolated, hypoxic environment with smaller populations (up to 42 during winterover) and much lower external microbial burden, resulting in heightened physiological and psychological stress (30). These conditions may dampen mucosal antibody responsiveness, causing valacyclovir’s effects to manifest instead in systemic markers such as IgG2. At South Pole, the profound suppression of recurrent EBV and HSV-1 reactivation may shift the immune system toward a more stable, low-inflammation state in which IgG2 contributes more prominently relative to placebo.

Our findings are not without limitations. In our study, we assessed total salivary immunoglobulins rather than viral specific antibodies (e.g., EBV VCA-IgG, EBV EA-IgG, HSV-1-specific IgG subclasses). Thus, we are unable to specifically distinguish whether EBV or HSV-1 or some other unrelated antigenic stimuli caused the reductions observed. Second, analyses performed in this study were on samples that had gone through their third freeze-thaw cycle: (1) original freezing of sample in Antarctica and subsequent thaw for processing for DNA extraction and saliva supernatant aliquoting, then refrozen; (2) thawing of aliquoted supernatants for cortisol and DHEA analysis in original research article, then refrozen; and (3) thawing for CRP and Immunoglobulin analysis, then refrozen. Additionally, these samples were originally collected between March and October 2020, with analyses for this study being conducted in late 2023 and early 2024, resulting in some saliva samples that were stored for at least 3 years in −80 °C. CRP has been shown to be stable for up to four freeze-thaw cycles (31), and up to five freeze-thaw cycles have had little effect on IgG stability (32). While the literature supports that freeze-thawing three times has likely had little effect on our sample outputs, the multi-year storage of our samples may have led to measurable reductions in certain analytes, particularly salivary IgA, which has been shown to have reductions of >10% after frozen storage of just 8 months or more (33). This could explain why—when comparing our IgA concentrations to published healthy references ranges—our values were markedly lower. Healthy adults have been shown to have salivary IgA levels ranging from 25–330 μg/mL (34), whereas expeditioners in this study exhibited IgA concentrations of 1, 981–6, 892 ng/mL (1.981–6.892 μg/mL). Therefore, the absolute values of IgA should be interpreted with that in mind.

Although Antarctic stations overlap well with spaceflight conditions, they cannot fully mimic the unique physiological and environmental challenges encountered during actual spaceflight missions. Microgravity and space-radiation exposure, alone and in combination with the other stressors, are difficult to replicate on Earth. Microgravity analogs in human subjects have produced mixed results: head down tilt bed rest has shown to not elicit strong immune or viral reactivation responses (35), whereas dry immersion bed rest has demonstrated extensive transcriptomic reprogramming of T cells toward a suppressed, naïve-like status (36). The strongest evidence for microgravity immune effects has been shown in cell culture models using clinostats (i.e. rotating wall vessels) which have shown clear functional changes in T-cells, macrophages, and NK cells (37). Furthermore, cell-culture studies have shown that simulated galactic cosmic radiation (GCRsim) and other space-relevant ionizing radiation types can directly trigger reactivation of latent viruses such as EBV and CMV (38, 39). Finally, while Antarctic expeditioners traverse long distances to reach their stations, they do not do so aboard a rocket, highlighting that ground-based analogs can approximate and inform, but not fully capture, the totality of stresses experienced during launch, transit, and life onboard spacecraft. As such, predictions about countermeasure efficacy made in Antarctic analogs remain provisional until validated in astronauts during missions on the ISS.

Collectively, these findings indicate that valacyclovir not only suppresses herpesvirus reactivation but also reshapes the immune landscape by reducing systemic inflammation and altering IgG subclass profiles. We hypothesize that, mechanistically, valacyclovir does not directly modulate these immune parameters, rather this effect is from directly preventing EBV and HSV-1 lytic (and presumably VZV) replication within infected cells, thus reducing persistent antigenic stimulation. Insights from this Antarctic analog are directly relevant to spaceflight, where astronauts face similar physiological and psychological stressors. Understanding how antiviral countermeasures stabilize immune function in extreme environments can inform strategies to protect crew health and performance during long-duration missions to the Moon, Mars, and beyond.

## Data Availability

The raw data supporting the conclusions of this article will be made available by the authors, without undue reservation.
