# Supplementary material for: Valacyclovir reduces inflammation and IgG subclass activation by suppressing herpesvirus reactivation in antarctic expeditioners
Source: Front Immunol. 2026 Oct 19;17:1957541. doi: 10.3389/fimmu.2026.1957541 (PMC13725367; doi:10.3389/fimmu.2026.1957541)
Supplement: Supplementary file 1 [file DataSheet1.pdf]

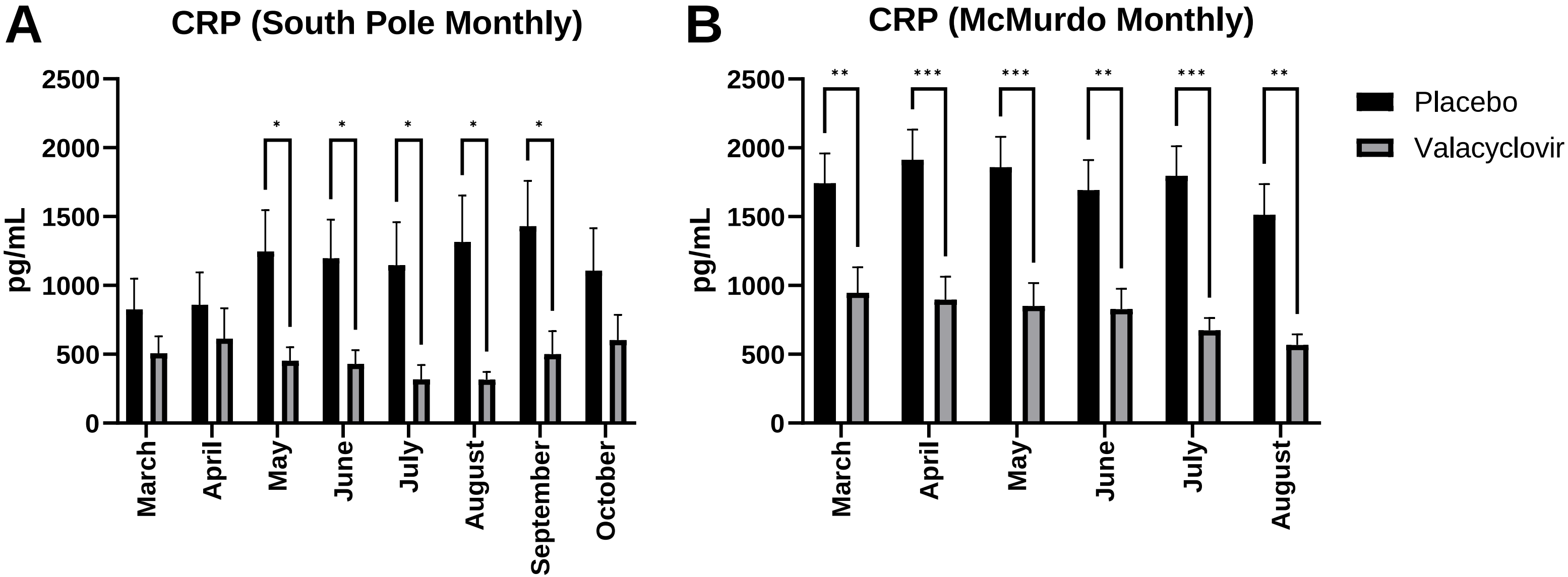

Supplementary Figure 1. Station-specific salivary CRP measurements in placebo and valacyclovir groups stratified by month. *p < 0.05, ** p < 0.01, *** p < 0.001
