# Supplementary material for: Valacyclovir reduces inflammation and IgG subclass activation by suppressing herpesvirus reactivation in antarctic expeditioners
Source: Front Immunol. 2026 Oct 19;17:1957541. doi: 10.3389/fimmu.2026.1957541 (PMC13725367; doi:10.3389/fimmu.2026.1957541)
Supplement: Supplementary file 2 [file DataSheet2.pdf]

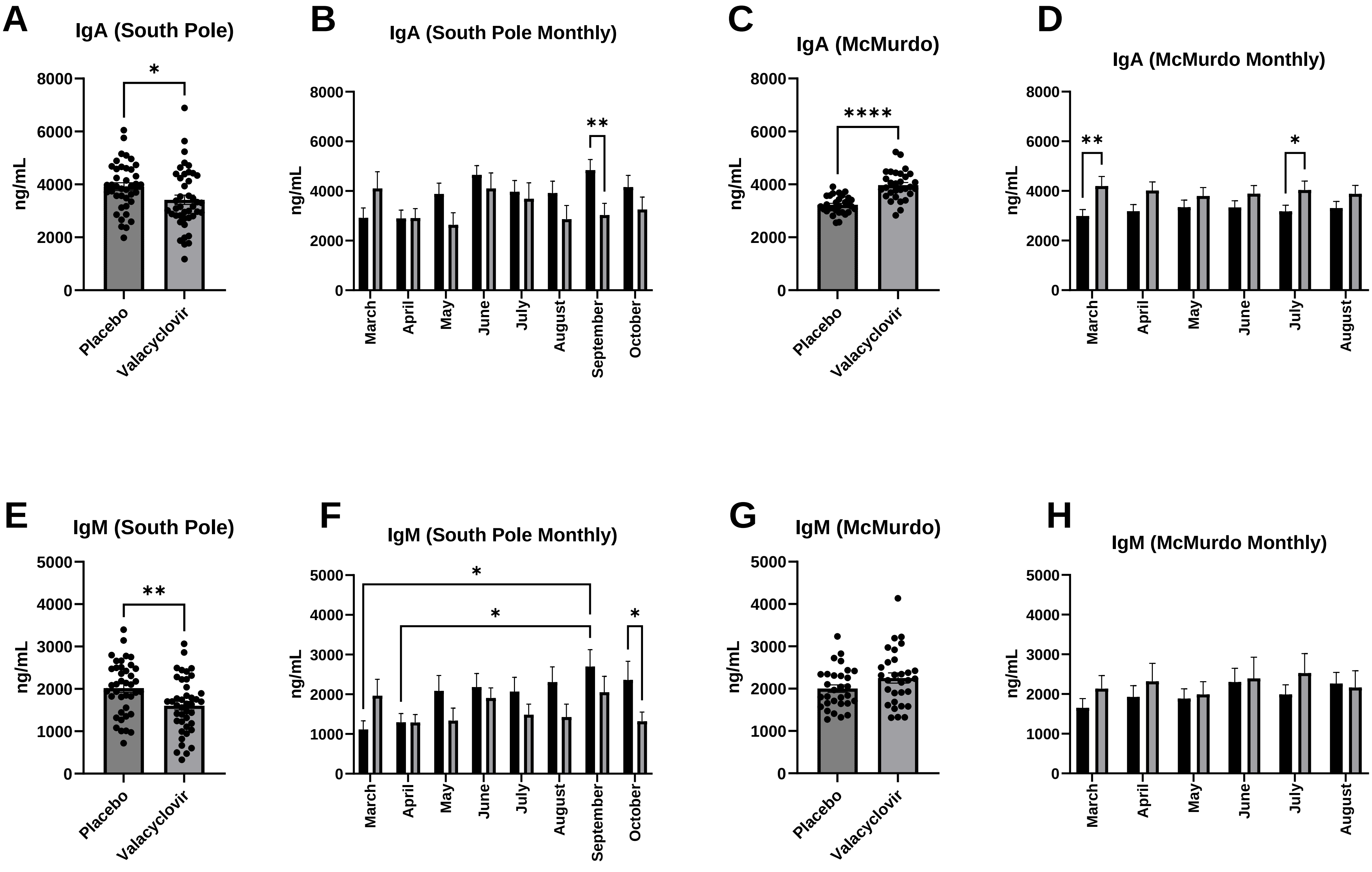

Supplementary Figure 2. Station-specific overall (A, C, E, G) and station-specific stratified by month (B, D, F, H) IgA and IgM measurements in placebo and valacyclovir groups.
***p < 0.05, ** p < 0.01, **** p < 0.0001**
