# Supplementary material for: Valacyclovir reduces inflammation and IgG subclass activation by suppressing herpesvirus reactivation in antarctic expeditioners
Source: Front Immunol. 2026 Oct 19;17:1957541. doi: 10.3389/fimmu.2026.1957541 (PMC13725367; doi:10.3389/fimmu.2026.1957541)
Supplement: Supplementary file 3 [file DataSheet3.pdf]

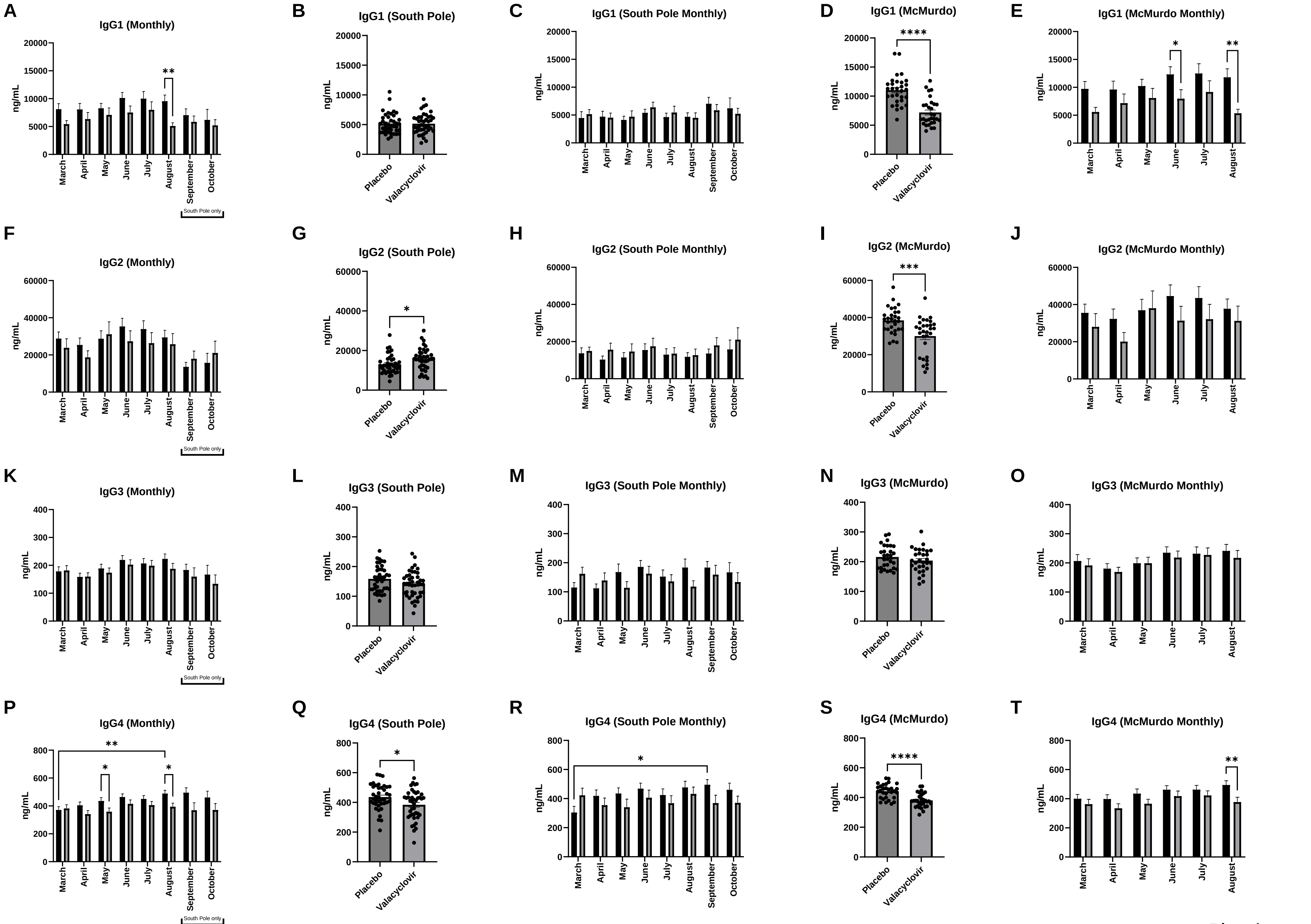

Supplementary Figure 3. Overall IgG1, IgG2, IgG3, and IgG4 concentrations stratified by month (A, F, K, P). Station-specific overall (B, D, G, I, L, N, Q, S) and station-specific stratified by month (C, E, H, J, M, O, R, T) IgG1-4 measurements in placebo and valacyclovir groups. *p < 0.05, ** p < 0.01, *** p < 0.001, **** p < 0.0001
